# AM1241 inhibits chondrocyte inflammation and ECM degradation through the Nrf2/HO-1 and NF-κB pathways and alleviates osteoarthritis in mice

**DOI:** 10.1186/s10020-024-01012-5

**Published:** 2025-01-10

**Authors:** Zhuan Zou, Songmu Pan, Changzheng Sun, Jiyong Wei, Yi Xu, Kaizhen Xiao, Jinmin Zhao, Ronghe Gu

**Affiliations:** 1 Department of Spine Surgery, The Fifth Affiliated Hospital of Guangxi Medical University, 89 Qixing Road, Nanning, Guangxi 530022 China; 2 Department of Pharmacy, Wenzhou Medical University, Wenzhou, Zhejiang 325000 China; 3 Department of Pharmacy, First Affiliated Hospital of Wenzhou Medical University, Wenzhou, Zhejiang 325000 China; 4 School of Basic Medical Sciences, Guangxi Medical University, No. 22, Shuangyou Road, Qingxiu District, Nanning, Guangxi 530021 China

**Keywords:** Osteoarthritis, AM1241, Lipopolysaccharide, Chondrocyte, Inflammation, Molecular docking

## Abstract

**Background:**

This study aimed to investigate the impact of AM1241 on lipopolysaccharide (LPS)-induced chondrocyte inflammation in mice and its potential mechanism for improving osteoarthritis (OA).

**Methods:**

The OA mice model was established employing the refined Hulth method. The impact of different concentrations of AM1241 on mice chondrocyte activity was detected using CCK-8. Changes in the levels of LPS-induced inflammatory factors and cartilage extracellular matrix (ECM) degradation in chondrocytes were determined by western blot, RT-qPCR, ELISA, and immunofluorescence assays, respectively. The specific action modes and binding sites of AM1241 with NEMO/IκB kinases (IKKs) in the NF-κB pathway and Keap1 protein in the Nrf2 pathway were predicted via molecular docking and molecular dynamics simulation, and the NF-κB and Nrf2 pathways were detected using western blot and immunofluorescence. In vivo, the impact of AM1241 on OA mice was analyzed through safranin-fast green staining, IHC staining, Mankin score, and microCT.

**Results:**

AM1241 inhibited the levels of LPS-induced transforming growth factor-β (TGF-β1), tumor necrosis factor-α (TNF-α), interleukin 6 (IL-6), matrix metalloproteinase-13 (MMP-13), and a disintegrin and metalloproteinase with thrombospondin motif 5 (ADAMTS-5) and diminished the degradation of type II collagen and Aggrecan. For the mechanism, AM1241 regulated the NF-kB and Nrf2/HO-1 signaling pathways by binding to NEMO/IKKβ and Keap1 target proteins and suppressed the activation of the NF-κB signaling pathway by activating the Nrf2 in chondrocytes. In vivo, AM1241 inhibited bone anabolism, mitigated articular cartilage hyperplasia and wear, and reduced the Mankin score in mice, thereby hindering the development of OA.

**Conclusion:**

AM1241 inhibited activation of the NF-κB signaling pathway via activating Nrf2. It suppressed the expression of inflammation factors and the degradation of ECM in vitro, and improved OA in mice in vivo, suggesting its potential as an effective drug candidate for the treatment of OA. The remarkable efficacy of AM1241 in alleviating murine OA positions it as a potential therapeutic strategy in the clinical management of OA diseases.

**Supplementary Information:**

The online version contains supplementary material available at 10.1186/s10020-024-01012-5.

## Introduction

Osteoarthritis ( OA ) is a common chronic joint degenerative disease caused by the degeneration and wear of articular cartilage and is considered to be an important clinical problem worldwide (Barnett 2018), closely related to age, gender, previous joint injury, obesity, and mechanical injury (Padilla-Martinez et al. 2018). Chondrocytes are the main source of extracellular matrix (ECM) in articular cartilage and can produce Aggrecan (a proteoglycan) and Type II collagen (type II collagen) (Krishnan et al. 2018). However, during OA development, ECM degradation products generated by tissue hydrolysis and destruction in chondrocytes can promote inflammatory responses, including the expression of cytokines and matrix metalloproteinases (MMPs) (Theocharis et al. 2019a, b). Following joint injury, a sudden increase in the levels of proinflammatory cytokines in the joint upregulates protease activity in the cartilage, enhancing the degradation of ECM by MMPs and cartilage aggregating proteoglycanase, which contributes to a decrease in type II collagen and Aggrecan, thereby compromising the mechanical strength of the tissue and leaving it vulnerable to further damage and degradation, resulting in a vicious cycle that facilitates disease progression (Theocharis et al. 2019a, b; Krishnan et al. 2018). Current medications for OA are symptom-based and can provide temporary relief from the disease, mainly focusing on alleviating pain and joint stiffness, while maintaining physical joint function. Nonetheless, they have many side effects (Latourte et al. 2020). Thus, there is an urgent need to identify a new targeted drug for OA treatment.

Lipopolysaccharide (LPS) is a critical proinflammatory factor associated with the pathogenesis of OA. Substantial studies with solid evidence indicated that LPS induces inflammatory damage and apoptosis in chondrocytes (Li et al. 2018; Huang et al. 2016). By binding to cell surface-expressed homologous receptor CD14 and Toll-like receptor 4, LPS activates multiple signaling cascades to drive the expression of the pro-inflammatory cytokine, including tumor necrosis factor-α ( TNF-α ), interleukin-1 ( IL-1 ) and interleukin-6 ( IL-6 ) (Lee et al. 2013). Therefore, the LPS-induced chondrocyte inflammation model can effectively expose the degree of cartilage damage in OA and can be used to establish an in vitro chondrocyte injury model.

Normal cartilage function depends on the regulation of various signaling pathways, which are also involved in regulating the onset and development of OA. NF-κB is a transcription factor with a broad range of roles and is crucial for the transcriptional regulation of chondrocyte matrix anabolic, decomposition of metabolism-related proteins, macrophage polarization, and inflammatory cytokine expression (Ahmed et al. 2017). Additionally, most OA patients have high expression of NF-κB, indicating that NF-κB may be the most critical regulator in OA (Lin et al. 2017). Previous studies have pointed out direct or indirect activation and inhibition between Nrf2 and NF-ĸB pathways (Kim et al. 2019). And Nrf2 can curb NF-κB activation and lessen the production of inflammatory mediators like TNF-α and MMPs (Ywac et al.; Dw et al. 2018), thereby suppressing inflammation and impairing degenerative disease, providing an interface between redox and anti-inflammatory responses. Therefore, we speculated that Nrf2 and NF-ĸB pathways may be the underlying mechanism for improving OA (Nadia et al. 2018; Marchev et al. 2017), and the screening for drugs that are both Nrf2 activators and NF-κB inhibitors without affecting physiological activity is also an essential direction for the treatment of OA.

AM1241 is an effective, typical, and selective CB2 receptor agonist. Earlier studies revealed that AM1241 can inhibit bone cancer-induced pain, reduce bone loss and lower the incidence of fractures caused by cancer (Lozano-Ondoua et al. 2010), promote the differentiation of RAW 264.7 cells into osteoblasts, and increase the expression of HO-1 and Nrf2 (Li et al. 2020). At the same time, researchers found that AM1241 attenuates myocardial ischemia-reperfusion injury in rats by enhancing Pink1/parkin-mediated autophagy (Liu et al. 2021), while Pink1/parkin is involved in chondrocyte death triggered by whole cell autophagy and mitochondrial autophagy (Shin et al. 2019), suggesting that AM1241 may have research value in treating OA. Furthermore, it has been reported that upregulation of the cannabinoid receptor CB2 can mitigate the inflammatory responses of OA and rheumatic arthritis (Richardson et al. 2008) and that CB2 activated by sea eggplant root extract mediates the anti-inflammatory effect of OA synovial cells (Mariano et al. 2022), indicating that AM1241 may have a protective effect for OA as CB2 receptor agonist. However, the impact of AM1241 in the treatment of OA remains unclear. Thus, we investigated the role of AM1241 in LPS-induced inflammatory responses and the degradation of the ECM in vitro, predicted the specific action mode and binding sites of AM1241 with NEMO/IκB kinases (IKKs) in the NF-κB pathway and Keap1 protein in the Nrf2 pathway by molecular docking and molecular dynamics simulations, and explored its mechanism of action in mice chondrocytes. Also, we identified the therapeutic effect of AM1241 on an OA mice model induced by a modified Hulth method in vivo.

## Materials and methods

### Reagents and antibodies

AM1241 (purity > 97%), type II collagenase were purchased from Thermo Fisher (Shanghai, China). Anti-type II collagen, Aggrecan, ADAMTSS, MMP-13, GAPDH, Histone H3 primary antibody, anti-P65, anti-Nrf2, anti-HO-1, anti-Iκb-α, anti-TGF-β1, anti-TNF-α, anti-IL-6, LPS, DMSO were purchased from Beyotime (Shanghai, China). DMEM/F12 with 10% fetal bovine serum (FBS) and 1% penicillin/streptomycin antibiotics and EDTA were purchased from Thermo Fisher (Shanghai, China).

### Primary mice chondrocytes culture

Mice below 10 days old were euthanized by overdosed sodium pentobarbital. Articular cartilage of mice was collected under sterile conditions using a dissecting microscope. The tissue was treated with 2 mg/mL (0.1%) type II collagen at 37 °C for 4 h. Subsequently, the digested cartilage tissue was inoculated in suspension in tissue culture flasks. The chondrocytes were grown in DMEM/F12 with 10% FBS and 1% penicillin/streptomycin antibiotics (Thermo Fisher Shanghai, China) at 37 °C. The culture medium was changed immediately after 24 h of incubation. When the fusion rate reached 80–90%, cells were collected via 0.25% trypsin EDTA (Thermo Fisher) Then, they were transferred into 10 cm culture plates at the appropriate density and cultured to P2-P3 for experiments. The chondrocytes were cultivated at 5% CO_2_ and 37 °C, and the complete medium was changed every other day.

### Animal model

A total of 28 C57BL/6 male mice (20–25 g) aged 10 weeks were purchased from Sipeifu Biotechnology Co., Ltd. The experiment was performed according to the Guide for the Care and Use of Laboratory Animals of the National Institutes of Health and approved by the Medical Ethics Committee of Nanning First People’s Hospital. The mice were kept in a controlled environment with a 12-hour light/dark cycle and free access to food and water. The mice OA model was established by a modified Hulth method that severed the anterior cruciate ligament and medial meniscus of the mice (Rogart et al. 1999). After preoperative fasting without water for 12 h and weighing, mice were anesthetized by intraperitoneal injection of 2% sodium pentobarbital (40 mg/kg), and the skin was incised about l cm long along the medial edge of the patellar ligament. After separation, the joint capsule was incised and the anterior cruciate ligament and medial meniscus were severed. Penicillin (500,000 U) was administered intramuscularly for 5 consecutive days after surgery to combat infection. Mice with arthrotomy without cutting the medial meniscus ligament on the left knee were conducted as a sham group. After surgery, the mice were randomly divided into sham-operated group, Hulth group, Hulth + 3 mg AM1241 (12 mg/ml) and Hulth + 9 mg AM1241 (36 mg/ml) group.

### Experimental design

In the in vitro study, chondrocytes from mice were treated with 100 ng/ml LPS alone or pretreated with AM1241 (5, 10 µM) for 4 h, followed by stimulation with 100ng/ml LPS for 12 h. The control group only changed the culture medium without treatment. Cells were harvested after 24 h of incubation. For in vivo evaluation, mice were subjected to the Hulth method as described above. After the successful establishment of the OA model, the groups were treated as follows: Hulth + 3 mg AM1241 group (3 mg/kg/day; once a week; 12 mg/ml AM1241 dissolved in 5% DMSO saline for five weeks in the joint cavity) and Hulth + 9 mg AM1241 group (9 mg/kg/day; once a week; 36 mg/ml AM1241 dissolved in 5% DMSO saline was injected for five weeks in the joint cavity). At the same time, equal amounts of 5% DMSO saline were administered to the sham group and the control group. Postoperatively, the animals were driven for 30 min daily for 5 weeks. After that, the animals were sacrificed and cartilage tissue specimens were collected for histological and MicroCT scan analysis.The experiment was approved by the Medical Ethics Committee of Nanning First People’s Hospital (2022-172-01).

### Cell viability assay

The impact of AM1241 on chondrocyte viability of mice was detected via a Cell Counting Kit-8 (CCK-8) kit (Beyotime). First, P2 chondrocytes were transferred to 96-well plates (2000 cells/well) for inoculation and incubated in different concentrations of AM1241 (0-250 µM) for 24/48 h. Before assay, 200 µL of DMEM/F12 medium containing 10 µL of CCK-8 reagent was added to each well and incubated for 2 h at 37 ℃ in the dark. Then the incubated medium was transferred to an enzyme-labeled plate and cultivated by measuring the absorbance at 450 nm using an enzyme marker. Five replicate wells were set up for each group, and the experiment was carried out five times.

### TGF-β1, TNF-α, and IL-6 determination

The expression levels of TGF-β1, TNF-α, and IL-6 were measured by ELISA kits (E-EL-0162c, E-EL-M0049c, E-EL-M0044c, Elabscience, Wuhan, China) according to the manufacturer’s instructions. The optical density was read at 450 nm with a Microplate Reader.

### Reverse transcriptase-polymerase chain reaction (RT-PCR)

Total RNA of chondrocytes treated with 100ng/ml LPS alone or in combination with different concentrations of AM1241 (0, 5, 10 µM) were extracted from 6-well culture plates using TRIzol (Beyotime). 1000 ng of total RNA was reverse transcribed to synthesize cDNA. For qPCR, 10 µl reaction volume in total was applied, including 5 µL of 2 × SYBR master mix, with 0.25 µL of each primer. RT-PCR parameters: 10 min 95 °C for 10 min followed by 15 s 95 °C for 15s and 60 °C 1 min for 40 circles with CFX96 real-time PCR system. Cycle threshold (Ct) values were collected and normalized to GAPDH levels. The relative mRNA levels of each target gene were calculated using the 2^−∆∆Ct^ method. All the primers were designed via NCBI primer blast (Table 1).

**Table 1:** List of primer sequences of mice for real-time PCR

Gene	Primer sequences(forward/reverse)
Aggrecan	5’ - AGGATGGCTTCCACCAGTGC − 3’ 5’ - TGCGTAAAAGACCTCACCCTCC − 3’
COL2A1	5’ - CCTGGCAAAGCTGGTGAG − 3’ 5’ - GCCAGGAAGTCCCTGGAA − 3’
ADAMTS-5	5’ - GAGGATTTATGTGGGCATCAT − 3’ 5’ - TGGAGGCCATCGTCTTCAAT − 3’
MMP-13	5’ - TGATGACATCAAGAAGGTGGTGAAG − 3’ 5’ - TCCTTGGAGGCCATGTGGGCCAT − 3’
TGF-β1	5’ - CGTCAGACATTCGGGAAGC − 3’ 5’ - CAGCCACTCAGGCGTATCA − 3’
TNF-α	5’ - TACTCCCAGGTTCTCTTCAAGG − 3’ 5’ - GGAGGCTGACTTTCTCCTGGTA − 3’
IL-6	5’ - GAGTTGTGCAATGGCAATTC − 3’ 5’ - ACTCCAGAAGACCAGAGCAG − 3’
GAPDH	5’ - GGGTGTGAACCACGAGAAAT − 3’ 5’ - CCACAGTCTTCTGAGTGGCA − 3’

### Western blot

Total proteins were extracted from chondrocytes using RIPA lysis buffer with 1 mM PMSF (phenylmethane sulfonyl fluoride). Next, they were separated on ice for 10 min and then centrifuged at 12,000 rpm and 4 °C for 15 min, followed by a measurement of protein concentration using the BCA protein analysis kit (Beyotime). Sodium dodecyl sulfate-polyacrylamide gel electrophoresis (SDS PAGE) was applied to separate 40ng protein which would be transferred to polyvinylidene difluoride membranes (Bio-Rad, USA). After being blocked by 5% skim milk for 1 h, the membranes were incubated with the antibodies of type II collagen, Aggrecan, Nrf2, MMP-13, ADAMTS-5, TGF-β1, TNF-α, IL-6, p65, Histone H3, and IκBα overnight at 4 °C, and subsequently incubated with the corresponding secondary antibodies at room temperature for 1 h. After washing twice with PBST and once with PBS (10 min/time), the blots were observed with electrochemiluminescence plus reagent (Invitrogen). Finally, the intensity of these blots was quantified using imagelab 3.0 software (Bio-Rad).

### Immunofluorescence

For type II collagen, Aggrecan immunofluorescence staining was employed. Chondrocytes were treated with 100 ng/mL LPS for 12 h, or pretreated with AM1241 (5 and 10 µM) for 4 h before being stimulated with 100 ng/mL LPS for 12 h. For p65 and Nrf2 staining, the duration of LPS treatment was shortened to 2 h. After treatment, samples were washed with PBS three times and permeabilized with PBS diluted with 0.1% Triton X-100 for 15 min. Chondrocytes were then blocked with 5% bovine serum albumin for 1 h at 37 °C, washed with PBS, and incubated overnight in a humid chamber at 4 °C with PBS-diluted primary antibodies: type II collagen, Aggrecan, p65 (1:200) and Nrf2 (1:400). The next day, plates were washed and incubated with Alexa-Fluor^®^ 488 labeling or Alexa-Fluor^®^ 594-conjugated secondary antibody (1:400) for 1 h at room temperature and labeled with DAPI for 5 min. Finally, five areas of each slide were randomly selected for microscopic observation using a fluorescence microscope (Olympus Inc., Tokyo, Japan) and fluorescence intensity was measured using Image J software 2.1 (Bethesda, MDUSA).

### MicroCT scan

After 6 weeks, the knee joints of mice were taken and immersed in 4% paraformaldehyde. The samples from each group were fixed and then scanned by micro-CT (Skyscan 1176, Bruker, Belgium) at a voltage of 50 kV, a current of 500 µA and a resolution of 18 μm per pixel. The region of interest (ROI) was set to the subchondral bone, and the bone trabecular parameters in this region were analyzed to assess the bone healing.

Three-dimensional anatomical structures including bone volume/total tissue volume (BV/TV), bone surface area/bone volume (BS/BV), trabecular thickness (Tb.Th), and separation (Tb.Sp) were calculated with CTAn (Bruker MicroCT, Kontich, Belgium). CTVox software (Bruker MicroCT, Kontich. Belgium) was utilized to reconstruct the 3D images.

### Histopathological analysis

After MicroCT, the knee was treated with a decalcification solution for 4 weeks. The tissue was dehydrated and embedded with paraffin. Sagittal sections in 6-µm thickness were cut along the knee joint, and each joint section was stained with fast green and safranin-O. IHC staining was applied to type II collagen, MMP-13. Slides were dewaxed and then stained with Fast Green (Beyotime) for 6 min, washed at 20˚C, dehydrated, and stained with Safranin O (Beyotime) for 3 min at 20˚C. For type II collagen and MMP-13 IHC staining, endogenous peroxidase in the tissue was first blocked with 3% H_2_O_2_. Then, the samples were treated with 2.5% hyaluronidase (Beyotime) for 1 h at 37˚C for epitope retrieval. The sections were blocked with fetal bovine serum for 1 h and incubated with type II collagen and MMP-13 monoclonal antibody at 37 °C for 4 h. Finally, the sections were counterstained with hematoxylin. The extent of cartilage lesions was assessed using the modified light microscopic cartilage assay evaluation criteria, the Mankin score, with a blinded evaluation. Disease course: 1–5: early stage; 6–9: intermediate stage; 10–14 late stage. Five mice per group were subjected to histomorphometric analysis.

### Molecular docking and molecular dynamics simulation

The 3D structure of the AM1241 compound was downloaded from the PubChem database and then imported into Chemdraw 3D for energy minimization using the MM2 module. The target protein crystal structures of Keap1 protein as well as the NEMO/IKKβ structures were downloaded from the PDB database and subsequently visualized separately using pymol. Next, the ligands were docked to the receptors using Autodock vina 1.1.2 and the docking results were analyzed after water removal, hydrogenation, charge calculation, and merging of non-polar hydrogens. After that, protein binding was visualized using Pymol and discovery studio software to analyze the specific binding sites and interaction forces of AM1241 with NEMO/IKKβ and Keap1 proteins. Molecular dynamics simulations were performed using the Gromacs software package, the physical conditions were then set to constant temperature (310 K), constant pressure (101 kPa), and periodic boundary conditions, and the TIP3P water model was employed to simulate the human environment in a 0.145 mol/L neutral sodium chloride solution. After the state of all environments was balanced, the screened compound target complex system was utilized to carry out a 50nsMD simulation, in which the confirmation storage calculation was performed every 10 ps. Then, compounds with docking fractions below the cut-off value were validated using the Gromacs embedded program and the root mean square deviation (RMSD), root mean square fluctuation (RMSF), the radius of gyration (Rg), and intermolecular hydrogen bond (H bond) from the visualized MD simulation results. Finally, the stability of AM1241 binding to its target protein was analyzed.

### Statistical analysis

The results were expressed as mean ± standard deviation. Statistical analysis was performed using SPSS statistical software program 20.0. Data were compared using a one-way analysis of variance (ANOVA) followed by Tukey’s test. Non-parametric data (e.g., Mankin scores) were analyzed using the Kruskal-wallish test. p-values less than 0.05 were considered a significance.

## Results

### Effect of AM1241 on chondrocyte viability

**Fig. 1:**
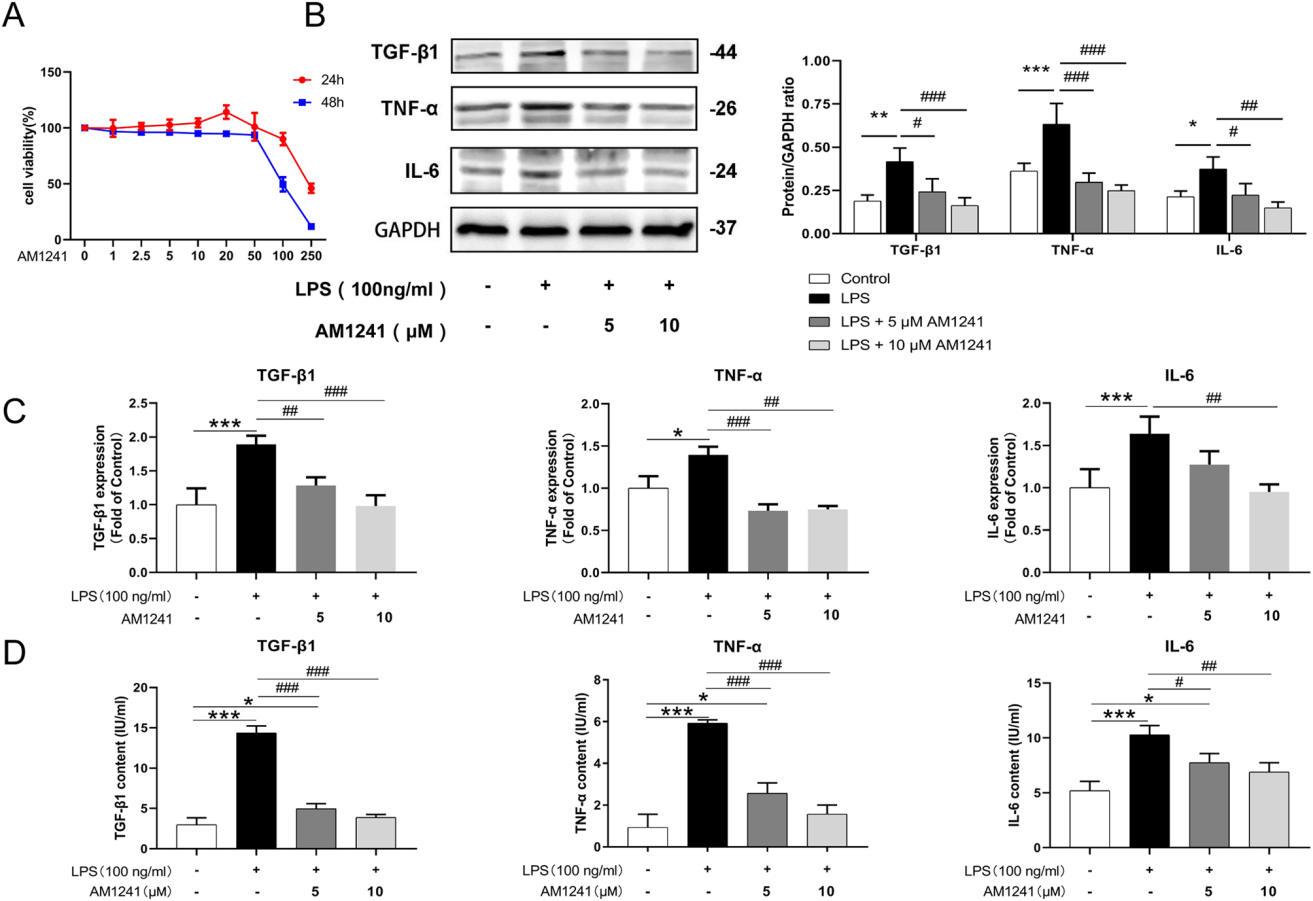
Effects of different concentrations of AM1241 on chondrocyte viability. Mice chondrocytes were taken and treated with different concentrations of AM1241 (0-250 µM) for 24 h/48 h. (**A**) The chondrocyte viability was determined to be the highest at 20 µM, and then the cell viability decreased. Cell experiments can be performed at concentrations below 20 µM. The effect of AM1241 on LPS-induced chondrocyte cytokines. Chondrocytes from mice were pretreated with 5 µM and 10 µM AM1241 for 4 h, and then stimulated with LPS for 12 h. (**B**) Western Blot analysis (left) and quantifications (right), (**C**) RT-PCR and (**D**) Elisa results showed that AM1241 could reduce the expression and secretion of TGF-β1, TNF-α, and IL-6 in chondrocytes. The data in the figure represent the mean ± standard deviation. Compared with the Control group, *P < 0.05, **P < 0.01, ***P < 0.001. Compared with the LPS group, ##*P* < 0.01, ###*P* < 0.001. Compared with LPS + 5 µM AM1241 group, &*P* < 0.05, &&*P* < 0.01, &&&*P* < 0.001; *n* = 3

After treating mice chondrocytes with increasing concentrations of AM1241 (0-250 µM) for 24 h and 48 h, the cell activity was measured using the CCK-8 assay. As shown in Fig. 1A, after 24 h of AM1241 treatment, the chondrocyte viability peaked at 20 µM and then decreased. This indicated that treatment of AM1241 with a concentration below 20 µM for 24 h and 48 h had no impact on mice chondrocytes. Thus, we used AM1241 at doses of 5 µM and 10 µM in subsequent experiments.

### AM1241 inhibits the expression and secretion of TGF-β1, TNF-α, and IL-6 in LPS-induced mice chondrocytes

To explore the role of AM1241 in the LPS-induced inflammatory response in mouse chondrocytes, RT-PCR, western blot, and ELISA were conducted. Results showed that LPS promoted the expression and secretion of the inflammatory factors TGF-β1, TNF-α, and IL-6, while AM1241 suppressed their expression in a dose-dependent manner (Fig. 1B-C).

### AM1241 attenuates LPS-induced ECM degradation in mice chondrocytes

**Fig. 2:**
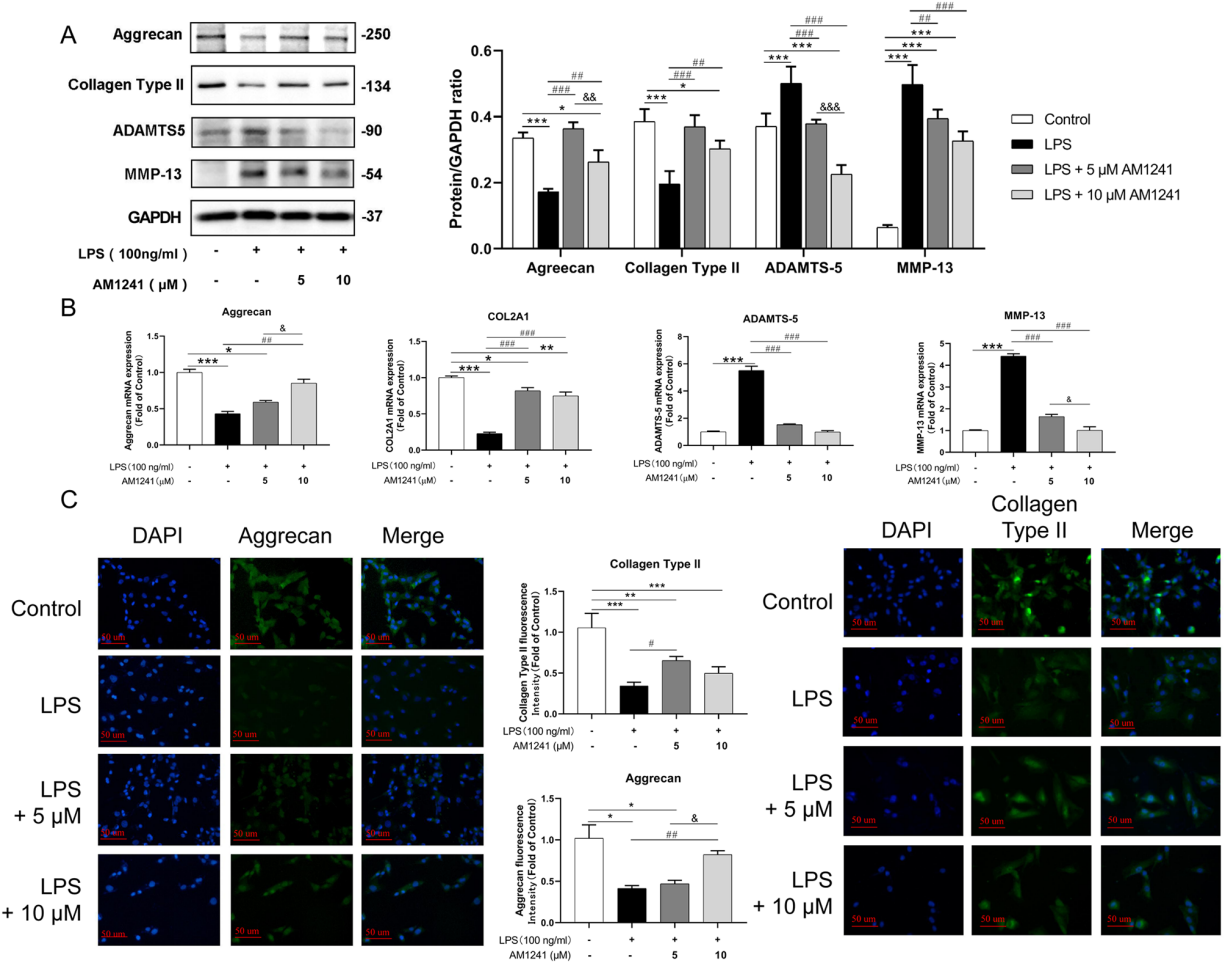
The effect of AM1241 on LPS-induced degradation of extracellular matrix in chondrocytes. Chondrocytes from mice were pretreated with 5 µM and 10 µM AM1241 for 4 h, and then stimulated with LPS for 12 h. (**A**) Western Blot analysis (left) and quantifications (right), and (**B**) RT-PCR showed the protein and mRNA expression of Aggrecan, type II collagen, ADAMTS-5 and MMP-13, respectively. (**C**) Cell immunofluorescence showed the expression of Aggrecan and type II collagen. The data in the figure represent the mean ± standard deviation. Compared with the Control group, *P < 0.05, **P < 0.01, ***P < 0.001. Compared with the LPS (100ng/mL) group, ##*P* < 0.01, ###*P* < 0.001; 5 µM AM1241 + LPS (100 ng/mL) group comparison, &*P* < 0.05, &&*P* < 0.01, &&&*P* < 0.001; *n* = 3

To evaluate how AM1241 affects LPS-induced ECM degradation, the western blot was carried out to determine the expression of type II collagen, Aggrecan, MMP-13 and ADAMTS5. As shown in Fig. 2A, LPS significantly impeded the synthesis of type II collagen and Aggrecan but enhanced the expression of MMP-13 and ADAMTS-5, which were important factors in promoting ECM degradation. And AM1241 pretreatment reversed LPS-induced ECM degradation in mice chondrocytes in a dose-dependent manner. In addition, the RT-PCR and immunofluorescence results on type II collagen and Aggrecan showed that LPS reduced the content of the two in mice chondrocytes, while AM1241 pretreatment increased the expression of type II collagen and Aggrecan. On the contrary, PT-PCR results illuminated that LPS promoted the expression of ADAMTS-5 and MMP-13, while AM1241 hindered the promotion, which was consistent with the outcomes of the western blot (Fig. 2B and C).

### Molecular docking and molecular dynamics simulations predict the binding of AM1241 to target proteins to regulate NF-κB and Nrf2/HO-1 pathways and the stability of the binding

To further explore the possible mechanism of the anti-inflammatory effect of AM1241, we initially hypothesized that AM1241 might inhibit the expression and secretion of inflammatory factors in chondrocytes by regulating NF-κB and Nrf2 signaling pathways. According to the results of molecular docking, we found that AM1241 could effectively bind to the active pocket of Keap1 protein, and its binding energy was − 8.2 kcal/mol (Figure S1A). Meanwhile, AM1241 could bind to the NEMO/IKKβ target protein with a binding energy of -7.4 kcal/mol (Figure S1B), indicating that the compound is capable spontaneous hydrogen bonding, hydrophobicity, and ion-π-conjugation interactions with the protein binding pocket. Further analysis of the three-dimensional interactions revealed that the compound could form hydrogen bonding interactions with the protein THR717. The benzene ring and purine structures in the compound were able to form ion-π conjugation interactions with HIS713 of the protein. And the hydrophobic functional groups in the compound could form hydrophobic interactions with ASP63, ALA64, GLN67, VAL709, ALA712 and HIS713 of the protein (Figure S1B). These interactions promoted ligand binding to the active pocket of the protein to form the complex. Based on this finding, we predicted that AM1241 may regulate NF-κB and Nrf2/HO-1 signaling pathways by binding to NEMO/IKKβ and Keap1 target proteins.

The stability of AM1241 binding to NEMO/IKKβ and Keap1 was comprehensively analyzed by molecular dynamics simulation. The RMSD trajectories of Keap1 and NEMO/IKKβ within 50 ns (Figure S2B, Figure S3C) indicated that after the initial fluctuation, both remained stable throughout the simulation. The plot of the RMSF results showed (Figure S2C) that fluctuations occurred in binding, and that regions of high fluctuations were observed consistent with the molecular docking results. The radius of rotation (Rg) indicated the size and denseness of the protein. The Rg value of Keap1 was 1.78 nm in the initial state (Figure S2D), without large fluctuations, and the system was stable up to 50 ns. The Rg for NEMO/IKKβ was 2.5 nm in the initial state (Figure S3B) and stabilized after 7 ns, then the system was stable. Also, the number of hydrogen bonds was calculated during the binding simulations. An average of 1–2 hydrogen bonds were observed in 50 ns simulations for Keap1 (Figure S2A), while an average of 1 hydrogen bond was found for NEMO/IKKβ (Figure S3A). All the RMSD, RMSF, Rg values and H-bond results suggested that AM1241 could bind to Keap1 protein and NEMO/IKKβ target proteins stably.

### AM1241 inhibits LPS-induced activation of NF-κB pathway in mouse chondrocytes

**Fig. 3:**
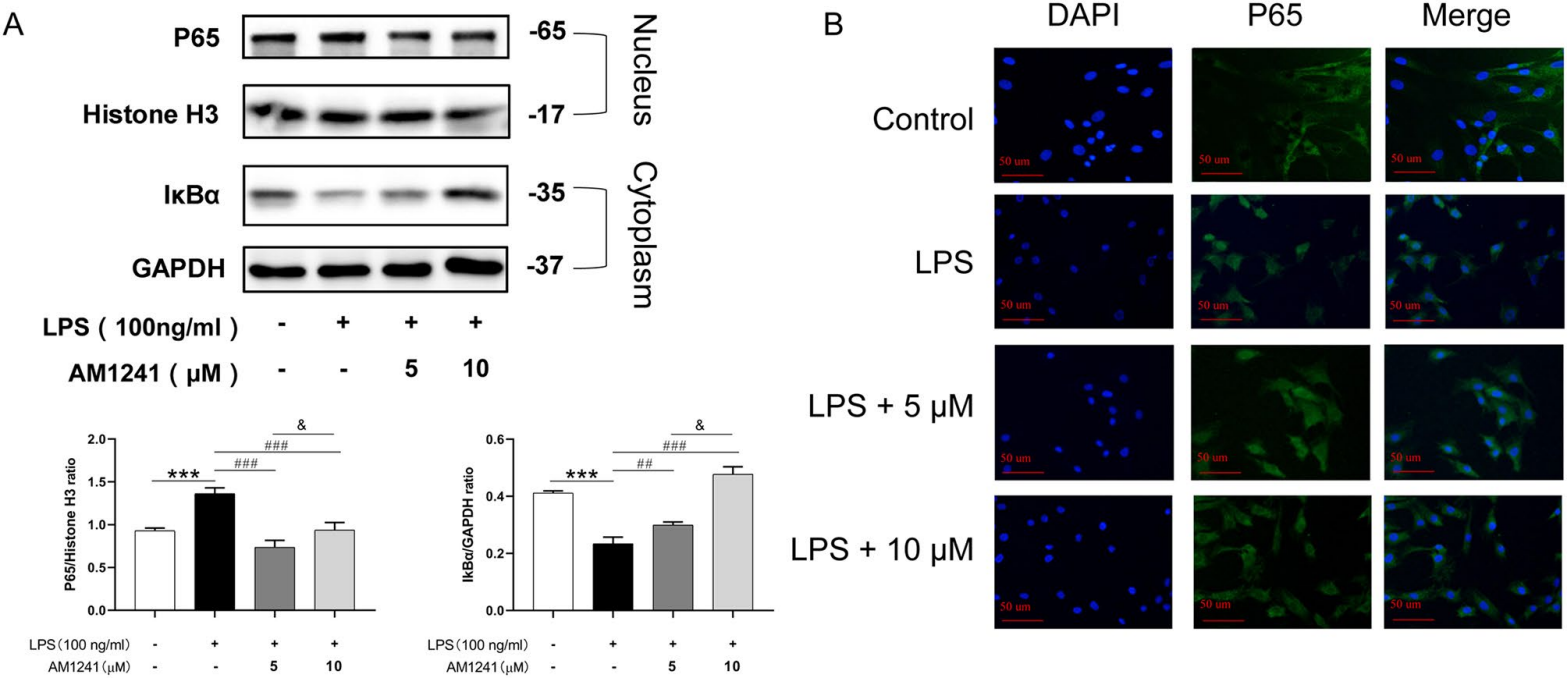
The effect of AM1241 on LPS-induced chondrocyte NF-κB pathway. Chondrocytes from mice were pretreated with 5 µM and 10 µM AM1241 for 4 h, and then stimulated with LPS for 2 h. (**A**) Western Blot analysis (upper) and quantifications (lower) showed the expressions of P65 and IκBα in the nucleus and cytoplasm, respectively. Green immunofluorescence (**B**) showed the localization of P65 in cells. The data in the figure represent the mean ± standard deviation. *P < 0.05 vs. LPS alone, *n* = 3. *P < 0.05 vs. the Control group. #*P* < 0.05, ##*P* < 0.01 vs. the LPS group; &*P* < 0.05 vs. the LPS + 5 µM AM1241 group; *n* = 3

To further verify the relationship between the anti-inflammatory effect of AM1241 and NF-κB, we investigated the activation level of the NF-κB pathway in mouse chondrocytes. Nuclear and cytoplasmic proteins were isolated from chondrocytes to compare the protein expression of IκBα in the cytoplasm and p65 in the nucleus. After LPS stimulation for 12 h, the expression of p65 in the nucleus and the degradation of IκBα in the cytoplasm both increased, while pretreatment with AM1241 for 4 h significantly reversed these changes (Fig. 3A). Furthermore, the translocation of p65 from the cytoplasm to the nucleus in mouse chondrocytes was assessed by immunofluorescence. As shown in Fig. 3B, in control chondrocytes, p65 was mostly located in the cytoplasm. After LPS stimulation for 12 h, p65 significantly translocated to the nucleus, whereas pretreatment with AM1241 for 4 h shows a trend of reduced green fluorescence in the nucleus.

### AM1241 activates the Nrf2/HO-1 pathway in mouse chondrocytes

**Fig. 4:**
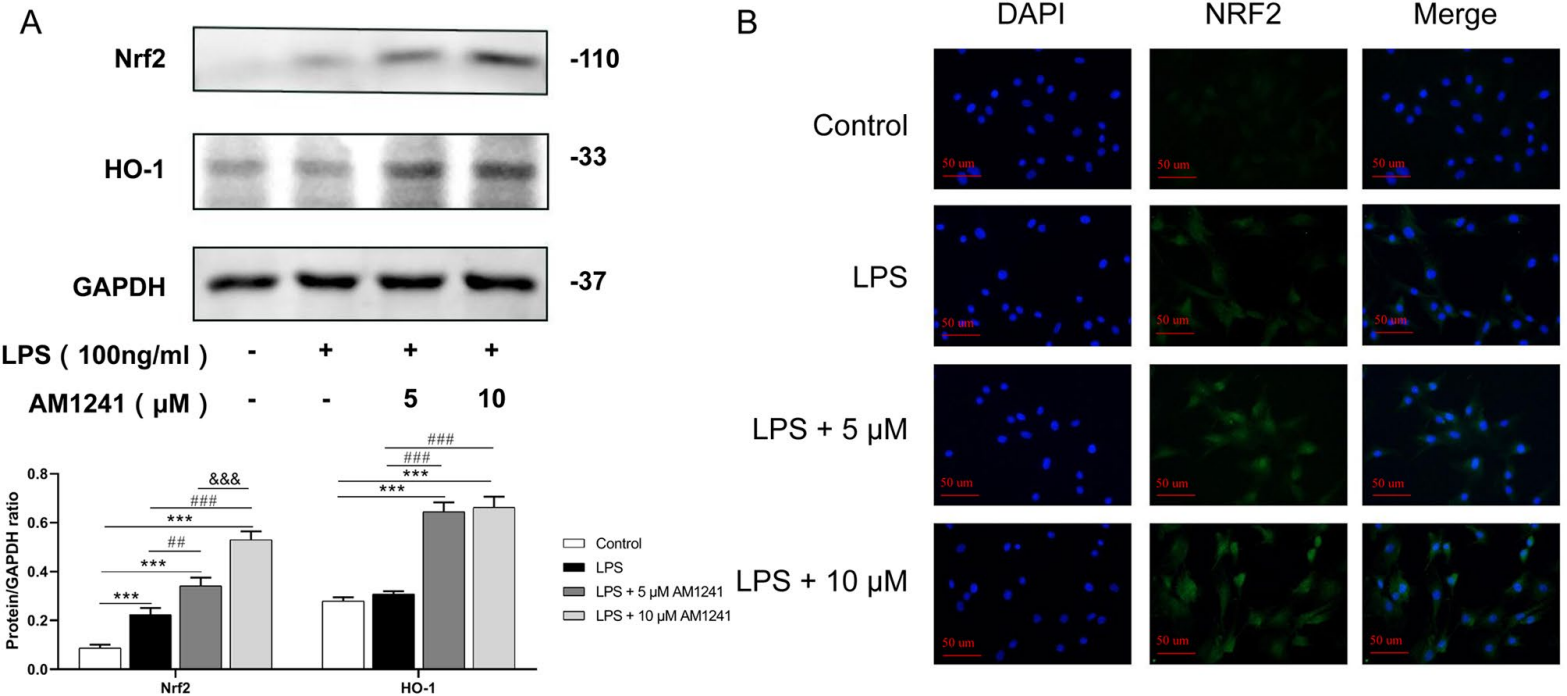
The effect of AM1241 on LPS-induced chondrocyte Nrf2 pathway. Chondrocytes from mice were pretreated with 5 µM and 10 µM AM1241 for 4 h, and then stimulated with LPS for 2 h. (**A**) Western Blot analysis (upper) and quantifications (lower) showed the expression of Nrf2. (**B**) Green immunofluorescence shows the localization of Nrf2 in cells. ***P < 0.001 vs. the Control group; ##*P* < 0.01, ###*P* < 0.001 vs. the LPS (100 ng/mL) group; &&&*P* < 0.001 vs. the 5 µM AM1241 + LPS (100 ng/mL) group; *n* = 3

The western-bolt results showed (Fig. 4A) that LPS stimulation promoted the expression of Nrf2 in chondrocytes, and AM1241 could further promoted the expression of Nrf2. Similarly, AM1241 promoted HO-1 expression in chondrocytes, but LPS treatment did not affect HO-1 expression. Moreover, the immunofluorescence staining results revealed that LPS stimulation enhanced the fluorescence intensity of Nrf2, indicating that LPS promoted Nrf2 expression in chondrocytes. Meanwhile, AM1241 further increased the intensity of Nrf2 fluorescence in LPS-induced mouse chondrocytes and Nrf2 accumulated from the cytoplasm to the nucleus, suggesting that AM1241 could activate the Nrf2 pathway and promote its nuclear translocation (Fig. 4B).

### AM1241 improves OA development in mice

**Fig. 5:**
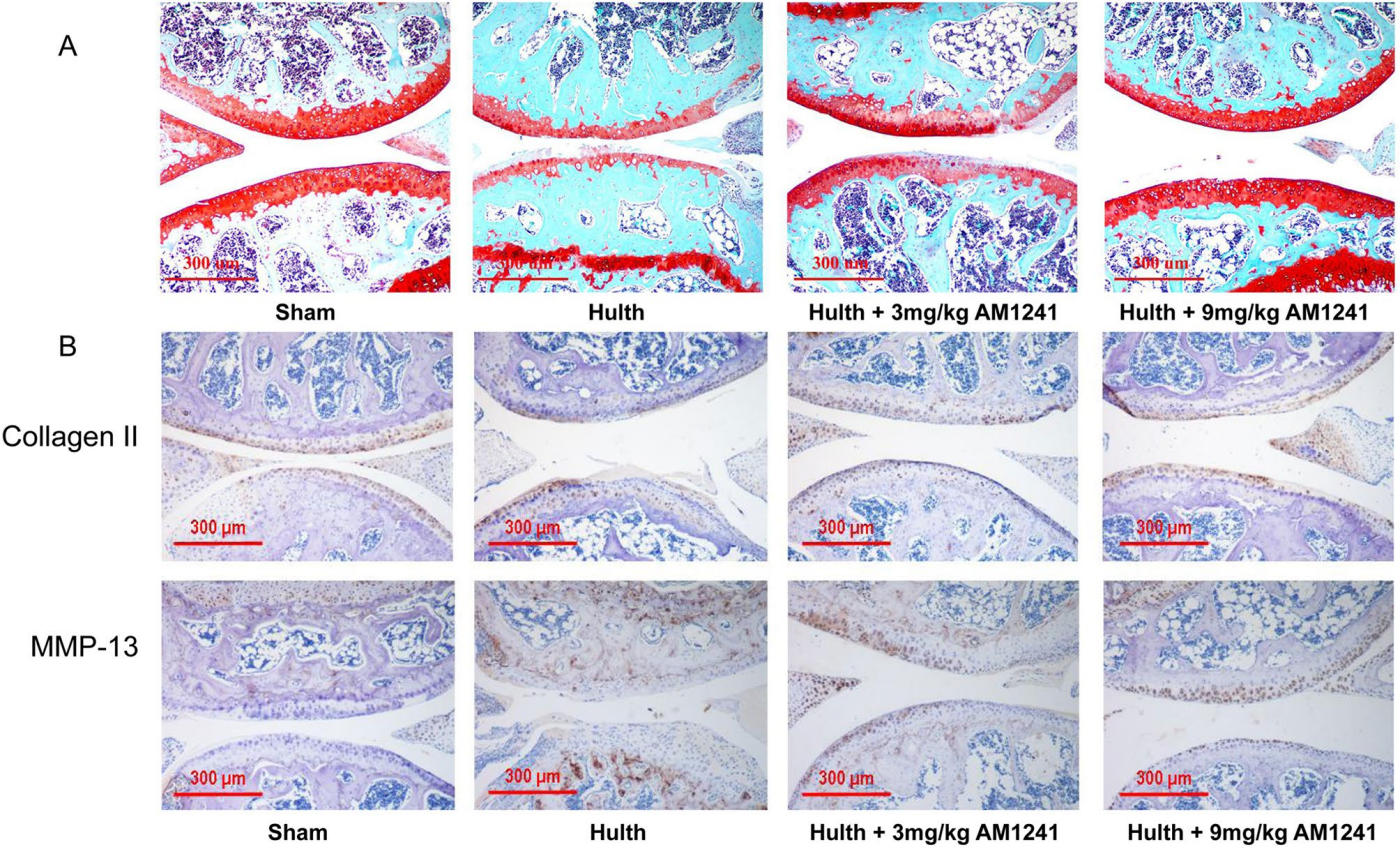
AM1241 ameliorates OA at the animal level. (**A**) Safranin-solid green staining of the knee joint. (**B**) IHC staining of the knee joint for collagen type II, MMP-13

**Fig. 6:**
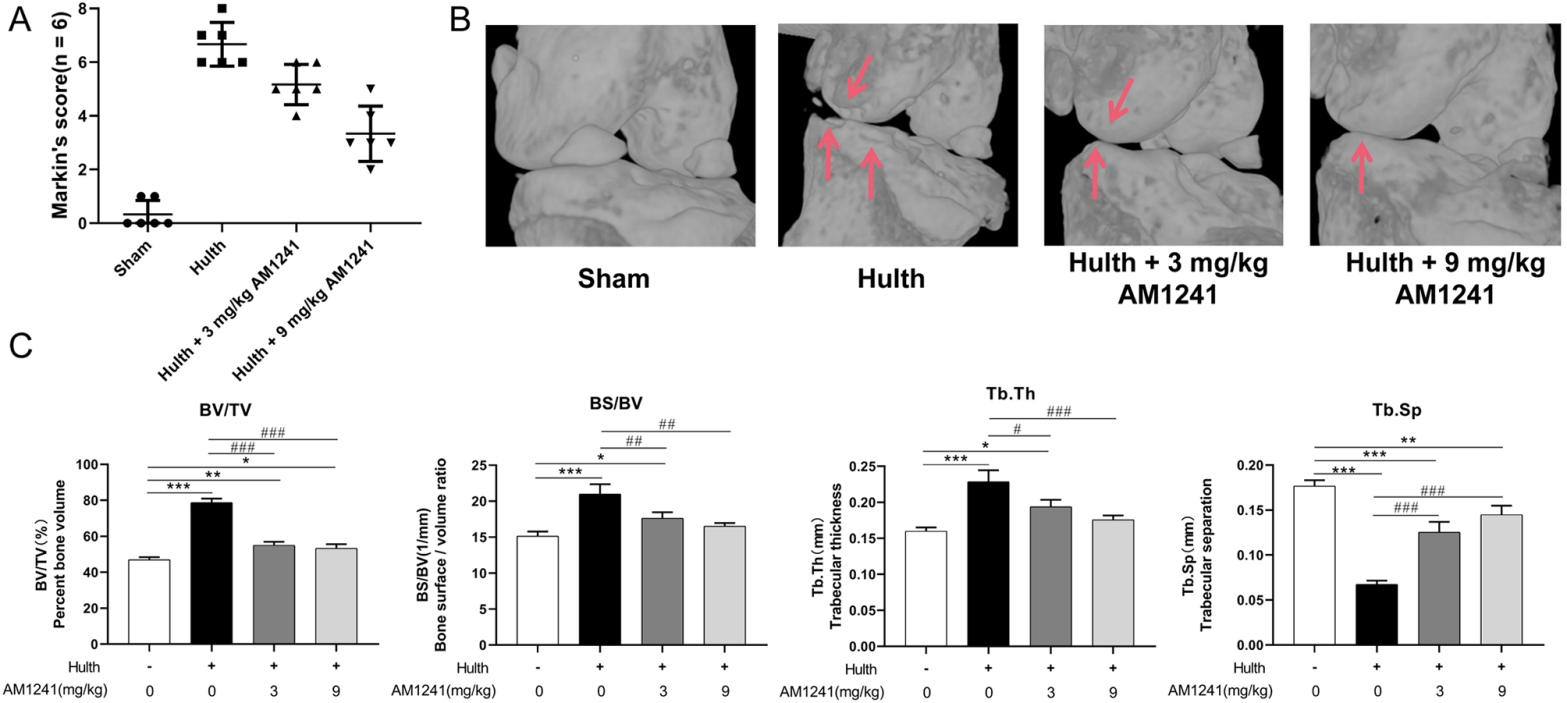
MicroCT general view and parameters. (**A**) The Mankin score; (**B**) Gross microCT image shows wear on the medial femoral condyle and medial tibial plateau surface. Arrow indicates abrasion on the medial femoral condyle and medial tibial plateau surface. (**C**) BV/TV (relative bone volume), BS/BV (bone surface area to bone volume ratio), Tb.Th (trabecular thickness), and Tb.Sp (trabecular separation) of the subchondral bone were analyzed via MicroCT, respectively. Figures represent mean ± standard deviation. *P < 0.05, **P < 0.01, ***P < 0.001 vs. Control group; ##*P* < 0.01, ###*P* < 0.001 vs. Hulth group; *n* = 3

As shown by safranin-solid green staining (Fig. 5A), the Hulth group presented cartilage erosion and significant cell reduction compared to the sham-operated group. In contrast, the AM1241-treated group showed smoother cartilage surfaces. The IHC staining results (Fig. 5B) suggested that type II collagen degradation induced by LPS in ECM was inhibited by AM1241 treatment. At the same time, the LPS-induced MMP-13 expression was elevated and then diminished after AM1241 treatment. The Mankin score showed (Fig. 6A; Table 2) a notably increased score in the Hulth group compared with the control group, indicating that the early Hulth model was successfully established. After AM1241 treatment, the Hulth + 3 mg/kg AM1241 group and Hulth + 9 mg/kg AM1241 group had considerably lower scores than the Hulth group, suggesting that AM1241 prominently improved OA in a dose-dependent manner. As shown in Fig. 6B, the postoperative medial femoral condyle and tibial plateau surface was severely worn. The AM1241 treatment group had lighter wear and the 9 mg/kg AM1241 treatment group had a better therapeutic effect. According to the microCT scan results (Fig. 6C), the articular BV/TV (relative bone volume), BS/BV (bone surface area bone volume ratio) and Tb.Th (bone trabecular thickness) were lessened in the postoperative AM1241 treatment group, while the Tb.Sp (trabecular separation) was increased, indicating that AM1241 inhibited bone anabolism, which in turn resulted in less articular cartilage proliferation and relatively reduced wear.

**Table 2:** Mankin’s score

Group	Score
Control	0.33 ± 0.51
Hulth	6.66 ± 0.81
Hulth + 3 mg/kg AM1241	5.16 ± 0.75
Hulth + 6 mg/kg AM1241	3.33 ± 1.03

## Discussion

In this study, we predicted the specific interaction modes and binding sites of AM1241 with NEMO/IKKβ in the NF-κB pathway and Keap1 protein in the Nrf2 pathway by molecular docking and molecular dynamics simulations and experimentally verified that AM1241 inhibits the regulatory effect of LPS on the NF-κB pathway in chondrocytes through activation of the Nrf2 pathway, thereby reducing LPS-induced inflammation and ECM degradation in chondrocytes. At the same time, in vivo studies illuminated that AM1241 improved the development of Hulth surgery-induced OA in mice and that higher concentrations of AM1241 had more protective effects on the bone joints.

OA is an aseptic inflammatory disease mediated by multiple inflammatory factors (Rahmati et al. 2017). Therefore, the study of inflammatory factors is one of the hot spots in the current research on the pathogenesis, injury characteristics, and therapeutic intervention of OA. In the study of Terkawi (Terkawi et al. 2022), the development of low-grade inflammation (LGI) in joints is closely related to OA. The characteristics of LGI include increased concentrations of non-specific inflammatory markers such as C-reactive protein (CRP), E-selectin, tumor necrosis factor-α (TNF-α), interleukin IL-1, IL-6, and vascular endothelial growth factor (VEGF). In the LPS-stimulated inflammatory response, IKK is activated (Chen et al. 2001), leading to degradation of IκBα, freeing and translocation of p65, and increased expression of TGF-β1, TNF-α, and IL-6. After stimulating chondrocytes by LPS, ADAMTS-5 and MMP-13 were activated due to the production of other cytokines and MMPs induced by inflammatory factors like TNF-α and IL-6 (Wang et al. 2018), thereby suppressing the expression of Aggrecan and type II collagen and promoting the degradation of ECM. It was also demonstrated in our experiments that LPS stimulation could dramatically elevate the expression of TGF-β1, TNF-α, IL-6, ADAMTS-5 and MMP-13 and facilitate ECM degradation. ECM mainly provides tensile support and impact resistance (Jaabar et al. 2022). ADAMTS-5 is the major aggregated proteoglycanase involved in cartilage degradation. And MMP13 is the central node of the cartilage degradation network and has great potential in inhibiting OA development (2015). In the present study, we found that AM1241 significantly inhibited the production of LPS-induced inflammatory mediator and ECM degradation at the protein and mRNA levels in a concentration-dependent manner.

In addition, the modulation of these pro-inflammatory mediators by AM1241 was associated with the promotion of the Nrf2 pathway and inhibition of the NF-κB pathway. By molecular docking and molecular dynamics simulations, we predicted that AM1241 may regulate the NF-κB and Nrf2/HO-1 signaling pathways by binding to NEMO/IKKβ and Keap1 target proteins, respectively. Computer simulations have revealed that Ginsenoside compound K exhibits strong binding affinity for IKK, and in vitro experiments have confirmed its potential to prevent osteoarthritis (Kang et al. 2016). It has been shown that Nrf2 can inhibit the activation of NF-κB (Kim et al. 2019), which promotes OA chondrocyte apoptosis (Choi et al. 2019), and that Nrf2/HO-1 signaling can alleviate the development of OA by suppressing the inflammasome (Yan et al. 2020; Chen et al. 2019). Additionally, studies have demonstrated that AM1241 can act on the Nrf2 pathway. The research of Li et al. pointed out that AM1241 can activate and accelerate the transport of Nrf2 to the nucleus, and inhibit cardiomyocyte fibrosis in an Nrf2-dependent manner (Li et al. 2016). In the study by Zhang et al., AM1241 ameliorated oxidative damage, protected skeletal muscle, and reduced apoptosis through Nrf2 signaling (Zhang et al. 2019). Some molecules targeting the Nrf2/HO-1 and NF-κB pathways have been shown to alleviate OA. For instance, puerarin (Chen et al. 2021) and myricetin (Pan et al. 2019) have been demonstrated in both in vivo and in vitro studies to inhibit chondrocyte ECM degradation by targeting the Nrf2/HO-1 and NF-κB pathways, thereby alleviating OA symptoms in mouse models. Similarly, our study further verified that AM1241 targets NEMO/IKKβ to inhibit the NF-κB pathway and Keap1 to activate the Nrf2/HO-1 pathway for the treatment of OA.

Based on in vitro studies, we established a mouse OA model using the Hulth surgery and injected AM1241 at various concentrations into the joint cavity of mice. MicroCT, IHC staining and safranin-fast green staining were then used to observe the wear of the knee joints of mice under different treatments, and Mankin was applied to assess the extent of cartilage lesions. The results showed that AM1241 had a protective effect on articular cartilage proliferation and wear, indicating that AM1241 treatment effectively repaired the damaged cartilage in OA mice. Although glucocorticoids (such as dexamethasone), hyaluronic acid (HA) or non-steroidal anti-inflammatory drugs (NSAIDs) are commonly used clinically to control pain and inflammation in the treatment of OA (Jones et al. 2019), the lack of stimulation of cartilage ECM recovery affects the therapeutic effect of OA. AM1241 can induce anti-inflammation by improving microglia phenotype and can modulate the release of inflammation-related factors in animal models of colitis, bile duct ligation, and asthma (Ma et al. 2018). Meanwhile, AM1241 can inhibit chondrocyte ADAMTS-5 and MMP-13 and promote cartilage ECM synthesis in LPS-induced OA. Therefore, the multifunctional AM1241 serves as an inhibitor of inflammation and MMPs, as well as a CB2 receptor agonist, can meet the needs of OA treatment. However, the clinical application of AM1241 is limited by several factors, including its short plasma half-life, limited oral bioavailability, and extensive plasma/albumin binding. In mice, the plasma half-life of AM1241 (25 mg/kg, iv) is 37 min. Following oral administration, the half-life more than doubles compared to intravenous injection, yet its oral bioavailability remains approximately 21% (Wood et al. 2013). Previous studies have encapsulated AM1241 in hydrogels for cranial defect repair (Ai et al. 2022). Enhancing AM1241’s in vivo half-life and targeting specificity will be key focuses for future research. Furthermore, determining the optimal dosing concentration of AM1241 in vivo is a critical factor that necessitates further exploration in future research efforts.

## Conclusion

AM1241 has the potential to stimulate chondrocyte proliferation and up-regulate cartilage-specific gene expression. Furthermore, AM1241 has anti-inflammatory effects by inhibiting LPS-induced abnormal up-regulation of TGF-β1, TNF-α and IL-6. In addition, AM1241 can promote the synthesis of cartilage matrix, inhibit cartilage hyperplasia, and mitigate cartilage wear, ultimately preventing the degradation of articular cartilage caused by LPS-induced OA. These findings suggest that AM1241 may be a critical drug for the effective treatment of OA.

## Electronic supplementary material

Below is the link to the electronic supplementary material.

Supplementary Material 1: Figure S1. Molecular docking of AM1241 with Keap1 and NEMO/IKKβ. (A) The detailed connection method between AM1241 and Keap1. (B) The detailed docking method between AM1241 and NEMO/IKKβ. AM1241 is brown, and the blue solid line represents the hydrogen bond distance. (C) Autodock-vina was used for semi-flexible docking and the obtained scores and interaction energy results are shown in the table

Supplementary Material 2: Figure S2. Molecular dynamics simulation of AM1241 with KEAP1. (A) The number of hydrogen bonds formed by AM1241 and KEAP1. (B) Protein RMSD frequency distribution during the 50 ns simulation period. (C) RMS fluctuation (RMSF, root mean square fluctuation). (D) The radius of gyration (Rg)

Supplementary Material 3: Molecular dynamics simulation of AM1241 with NEMO/IKKβ. (A) The number of hydrogen bonds formed by AM1241 and NEMO/IKKβ. (B) The radius of gyration (Rg). (C) Protein RMSD frequency distribution during the 50 ns simulation period

## Data Availability

All data generated or analyzed during this study are included in this article. The datasets used and/or analyzed during the current study are available from the corresponding author on reasonable request.

## Abbreviations

LPS: Lipopolysaccharide
OA: Osteoarthritis
ECM: Extracellular matrix
TNF-α: Tumor necrosis factor-α
IL-6: Interleukin 6
MMP-13: Matrix metalloproteinase-13
ADAMTS-5: A disintegrin and metalloproteinase with thrombospondin motif 5
Nrf2: Nuclear factor erythroid 2-related factor 2
MMPs: Matrix metalloproteinases
IL-1: Interleukin-1
CCK-8: Cell Counting Kit-8
RT-PCR: Reverse transcription-polymerase chain reaction
ANOVA: A one-way analysis of variance
