# Supplementary figures and images for: AM1241 inhibits chondrocyte inflammation and ECM degradation through the Nrf2/HO-1 and NF-κB pathways and alleviates osteoarthritis in mice
Source: Mol Med. 2025 Jan 10;31:9. doi: 10.1186/s10020-024-01012-5 (PMC11721480; doi:10.1186/s10020-024-01012-5)

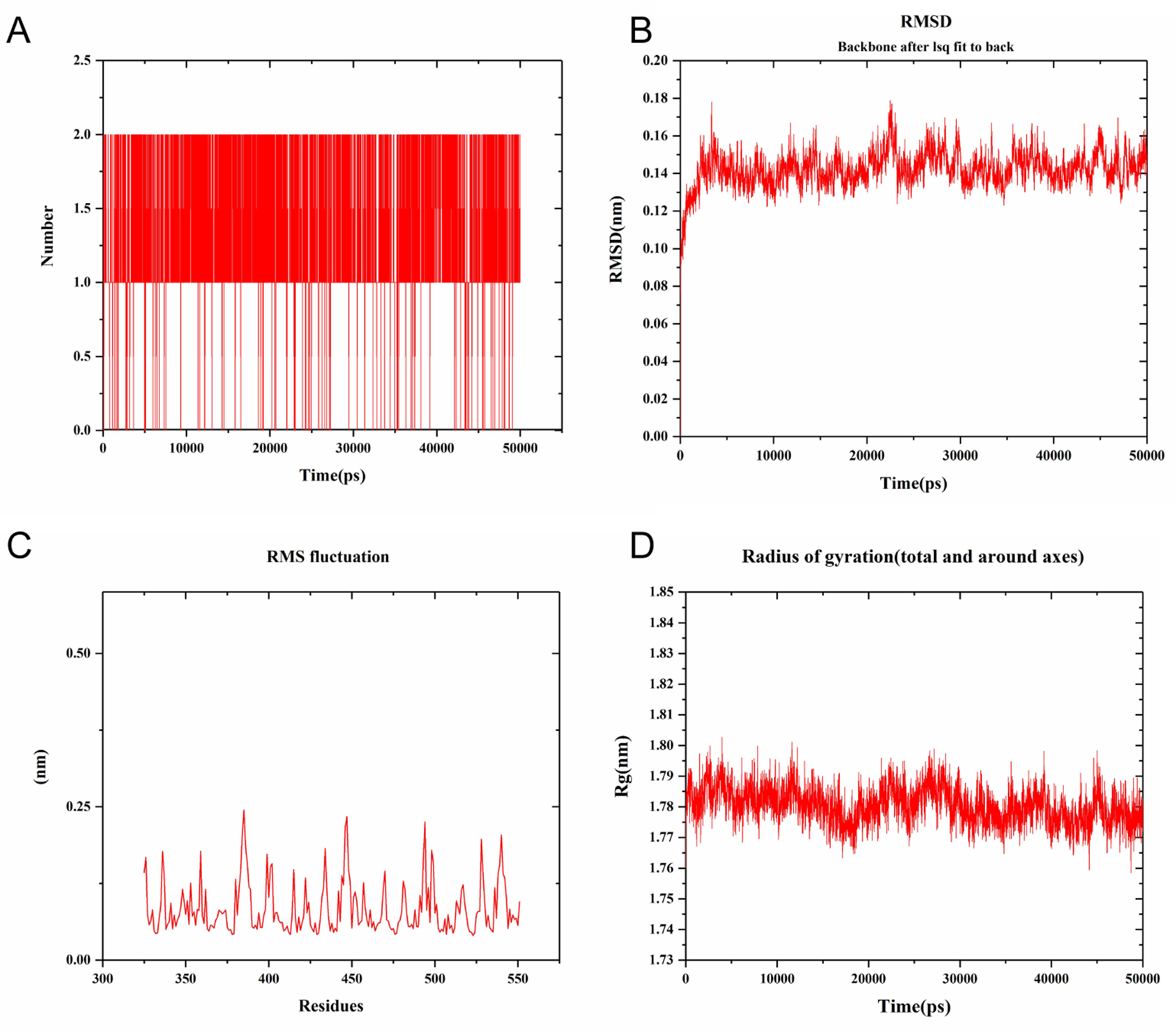

Supplement: Supplementary file 1 — Supplementary Material 1: Figure S1. Molecular docking of AM1241 with Keap1 and NEMO/IKKβ. (A) The detailed connection method between AM1241 and Keap1. (B) The detailed docking method between AM1241 and NEMO/IKKβ. AM1241 is brown, and the blue solid line represents the hydrogen bond distance. (C) Autodock-vina was used for semi-flexible docking and the obtained scores and interaction energy results are shown in the table [file 10020_2024_1012_MOESM1_ESM.tif]

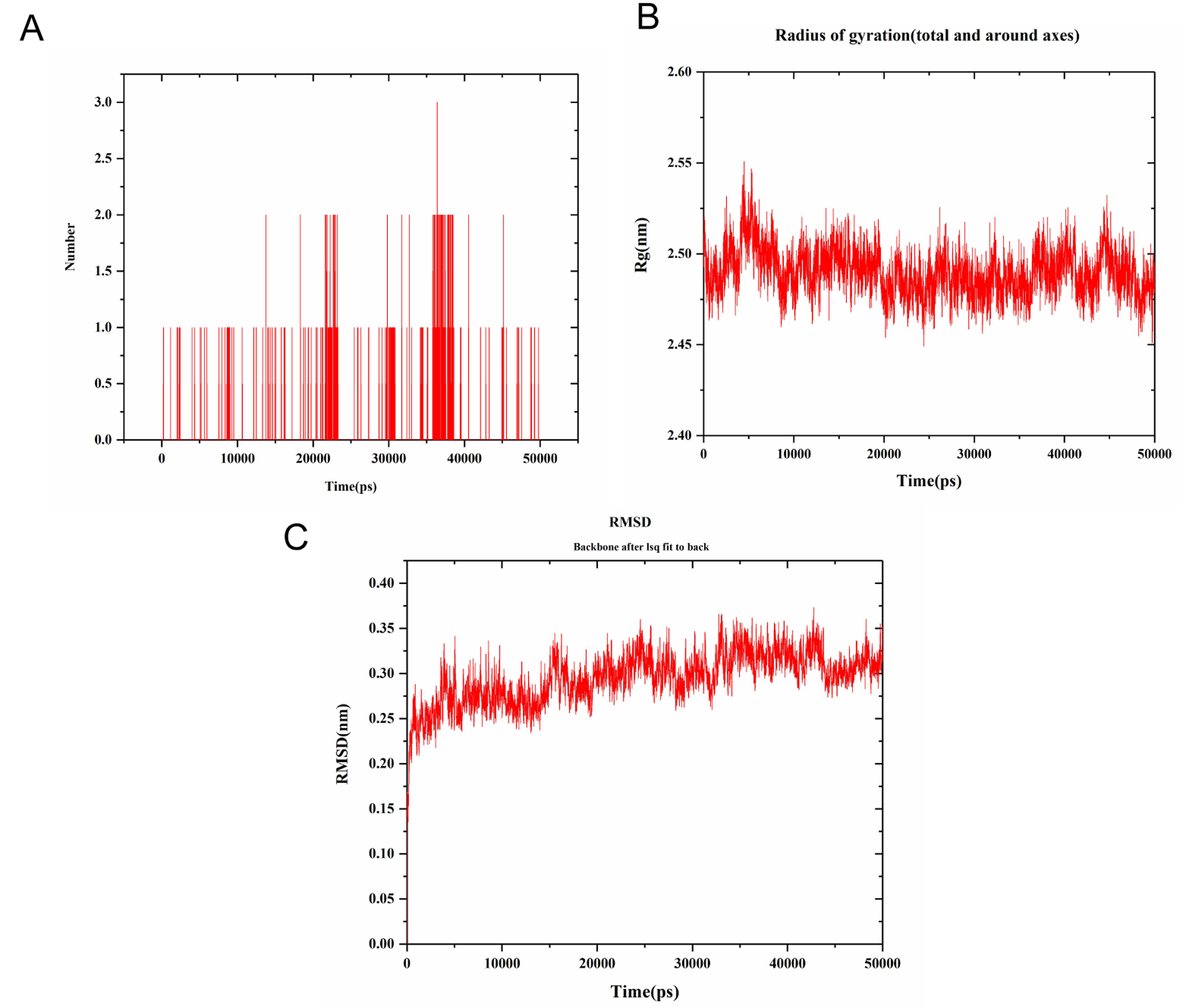

Supplement: Supplementary file 2 — Supplementary Material 2: Figure S2. Molecular dynamics simulation of AM1241 with KEAP1. (A) The number of hydrogen bonds formed by AM1241 and KEAP1. (B) Protein RMSD frequency distribution during the 50 ns simulation period. (C) RMS fluctuation (RMSF, root mean square fluctuation). (D) The radius of gyration (Rg) [file 10020_2024_1012_MOESM2_ESM.tif]

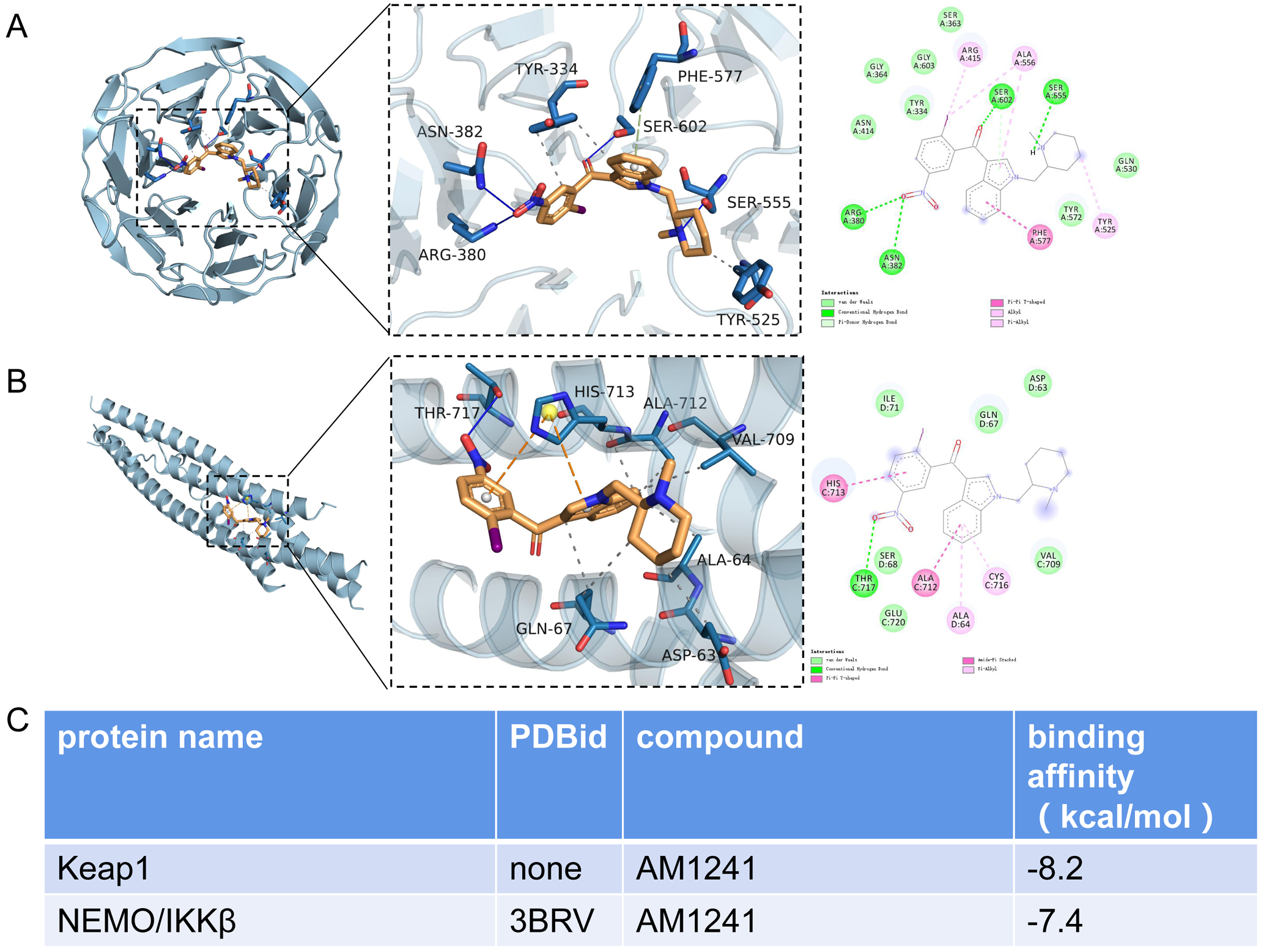

Supplement: Supplementary file 3 — Supplementary Material 3: Molecular dynamics simulation of AM1241 with NEMO/IKKβ. (A) The number of hydrogen bonds formed by AM1241 and NEMO/IKKβ. (B) The radius of gyration (Rg). (C) Protein RMSD frequency distribution during the 50 ns simulation period [file 10020_2024_1012_MOESM3_ESM.tif]
